# Immune-mediated renal injury and cardiometabolic risk in IgA nephropathy: clinical evidence on telitacicept from a scoping review

**DOI:** 10.3389/fnut.2026.1790988

**Published:** 2026-03-17

**Authors:** Li Zheng, Weina Zhang, Chumeng Yang, Yatong Zhang, Huiling Qi, Changhai Fu, Aijun Zhai

**Affiliations:** 1 Department of Pharmacy, Beijing Genertec Aerospace Hospital, Beijing, China; 2 Department of Pharmacy, Beijing Hospital, National Center of Gerontology, Institute of Geriatric Medicine, Chinese Academy of Medical Sciences, Beijing, China; 3 Department of Nephrology, Beijing Genertec Aerospace Hospital, Beijing, China; 4 Department of Geriatrics, Beijing Genertec Aerospace Hospital, Beijing, China

**Keywords:** immunoglobulin A nephropathy, telitacicept, 24-h urinary protein, estimated glomerular filtration rate, nutritional strategies, cardiorenal axis

## Abstract

**Background:**

Immunoglobulin A nephropathy (IgAN) is not only the most common primary glomerular disease but also a chronic inflammatory condition associated with increased cardiometabolic risk through the cardiorenal axis. Persistent proteinuria and progressive renal dysfunction are linked to adverse cardiovascular and metabolic outcomes and may complicate the delivery of long-term lifestyle and nutritional risk–modifying strategies. Telitacicept, a dual BAFF/APRIL inhibitor, has emerged as a targeted immunomodulatory therapy for IgAN, yet the clinical evidence remains heterogeneous.

**Methods:**

We conducted a scoping review of clinical studies evaluating telitacicept in biopsy-confirmed IgAN. PubMed, Embase, the Cochrane Central Register of Controlled Trials, and ClinicalTrials.gov were searched from inception to 2 January 2026. Randomized and observational studies reporting renal outcomes were included. When available, cardiometabolic- and nutrition-related variables (e.g., blood pressure, lipid profile, uric acid, body weight/BMI, inflammatory markers, and lifestyle/nutritional counseling) were also captured.

**Results:**

Twenty-four studies were identified, including one randomized controlled trial, 16 observational studies, and seven case reports/series. Across studies, telitacicept consistently reduced proteinuria (approximately 49–87%) while maintaining stable estimated glomerular filtration rate. Additional reported benefits included improvements in serum albumin-likely reflecting reduced urinary protein loss—and glucocorticoid-sparing effects. The therapy was generally well tolerated, with predominantly mild adverse events. Notably, cardiometabolic and nutritional endpoints were inconsistently reported across the current literature, limiting definitive conclusions regarding these outcomes.

**Conclusion:**

Current evidence suggests that telitacicept may offer a promising targeted therapeutic option for IgAN by achieving sustained proteinuria reduction and renal function stabilization. From a clinical practice perspective, improved renal and inflammatory control may facilitate the implementation of long-term nutritional and metabolic risk–modifying strategies in high-risk IgAN populations; however, direct evidence linking telitacicept to cardiometabolic or nutritional endpoints remains scarce. Larger, long-term randomized studies incorporating prespecified cardiometabolic and nutritional outcomes are warranted.

## 1 Introduction

Immunoglobulin A nephropathy (IgAN) is the most common primary glomerular disease worldwide and a leading cause of kidney failure in young adults, with marked geographic variation in disease burden (1–3). The pathological hallmark of IgAN is the mesangial deposition of immune complexes predominantly containing galactose-deficient IgA1 (Gd-IgA1), which initiates immune activation, inflammation, and progressive renal injury (1).

Beyond progressive renal injury, IgAN has increasingly been recognized as a systemic disease characterized by chronic inflammation and elevated cardiometabolic risk. Persistent proteinuria and declining renal function are strongly associated with endothelial dysfunction, metabolic disturbances, and accelerated cardiovascular disease. Although nutritional strategies, such as sodium restriction, cardioprotective dietary patterns, and metabolic risk optimization, are central to cardiometabolic disease prevention, their effectiveness in IgAN is often constrained by ongoing immune-driven renal injury. Consequently, therapeutic approaches that stabilize renal function and attenuate inflammatory burden are essential prerequisites for successful nutritional and cardiometabolic management in this population.

The pathogenesis of IgAN is most widely explained by the “multi-hit” hypothesis, in which elevated circulating levels of hinge-region galactose-deficient IgA1 represent a central initiating abnormality (4, 5). Subsequent formation of IgG and IgA autoantibodies against exposed N-acetylgalactosamine epitopes on Gd-IgA1, together with the intrinsic tendency of Gd-IgA1 to self-aggregate and bind serum proteins, promotes the generation of circulating immune complexes (6). Deposition of these large, charged complexes in the glomerular mesangium leads to mesangial cell activation and local inflammatory responses, ultimately driving renal damage (7). Among the upstream regulators of Gd-IgA1 production, B-cell activating factor (BAFF, also known as B-lymphocyte stimulator [BLyS]) and a proliferation-inducing ligand (APRIL) play critical roles by promoting aberrant mucosal B-cell activation and excessive IgA synthesis (8, 9). Accordingly, therapeutic strategies targeting B-cell–mediated pathways, particularly the BAFF/APRIL axis, have emerged as promising approaches in IgAN.

Telitacicept is a recombinant fusion protein that simultaneously neutralizes BAFF and APRIL and has been approved in China for the treatment of active systemic lupus erythematosus based on demonstrated efficacy and safety (10–12). In recent years, telitacicept has also been explored in patients with IgAN, with preliminary studies suggesting potential benefits in reducing proteinuria and stabilizing renal function. However, the available evidence remains limited and heterogeneous, encompassing studies with diverse designs, populations, interventions, outcome measures, and follow-up durations.

Given the early and exploratory nature of the existing literature, a scoping review approach is appropriate to comprehensively map and characterize the current evidence rather than to perform quantitative synthesis. Therefore, this scoping review aims to systematically identify and summarize the available studies on telitacicept in IgAN, delineate key study characteristics and reported outcomes, and identify knowledge gaps to inform future research and clinical trial design.

## 2 Methods

The review was guided by the methodological framework proposed by Arksey and O’Malley, with consideration of subsequent methodological refinements for scoping reviews. Reporting of this scoping review adheres to the Preferred Reporting Items for Systematic Reviews and Meta-Analyses extension for Scoping Reviews (PRISMA-ScR). The PRISMA-ScR checklist is provided in the Appendix 1. Because PROSPERO currently does not accept scope review, this study was not prospectively registered.

### 2.1 Search strategy and selection criteria

We searched PubMed, Embase, the Cochrane Central Register of Controlled Trials, and the ClinicalTrials.gov from database inception up to 2 January 2026, and screened the reference lists of relevant systematic reviews for additional trials. The search was conducted using a combination of controlled vocabulary (e.g., MeSH) and free-text terms. The search terms included “iga nephropathy,” “RC18,” “telitacicept” and “TACI-Fc” (Appendix 2).

Studies were included in this analysis if they met the following eligibility criteria: (1) study type: Quantitative studies, primarily including clinical trials and observational studies. Given the early and evolving clinical experience with telitacicept in IgA nephropathy, illustrative case reports and case series were intentionally included to capture emerging treatment patterns, responses in special populations, and potential safety signals. Evidence derived from case-based reports was considered exploratory and hypothesis-generating rather than confirmatory; (2) Patients with biopsy-confirmed IgAN who received telitacicept treatment; (3) Outcome Measures: Indicators that could be accurately measured and assessed in clinical studies and were applicable for evaluating changes in disease status following telitacicept treatment, such as 24-h urinary protein quantity (24hUPQ), serum creatinine (Scr), urine albumin-to-creatinine ratio (UACR), urine protein-to-creatinine ratio (UPCR), plasma albumin (Alb), urine red blood cell counts (URBC), and estimated glomerular filtration rate (eGFR). We excluded conference, abstracts, studies for which full text was unavailable, and studies not published in English. In addition to renal outcomes, we attempted to extract cardiometabolic- and nutrition-related variables when reported, including blood pressure, lipid profile, uric acid, glycemic indicators, body weight/BMI, inflammatory markers, cardiovascular events, and any documentation of lifestyle or nutritional counseling.

### 2.2 Study selection and data extraction

Initially, duplicate records were removed using EndNote software. Two researchers (CMY and WNZ) then independently screened the studies based on the inclusion and exclusion criteria. After completing the screening, the results were cross-checked, and any discrepancies were resolved through discussion. Subsequently, two researchers (HLQ and CHF) independently extracted data, including the first author, publication year, country of publication, study type, sample size, male-to-female ratio, intervention measures, intervention duration, outcome indicators, and treatment effects. After data extraction, a third researcher (LZ) verified the data, and any disagreements were resolved through discussion.

Consistent with the objectives of a scoping review, extracted data were synthesized using a structured descriptive and narrative approach rather than quantitative pooling. Study characteristics and outcomes were summarized and organized according to study design, patient population, treatment duration, and outcome domains. Given the heterogeneity in study designs, populations, and outcome definitions, no formal meta-analysis was performed, and heterogeneity was explored qualitatively.

## 3 Results

### 3.1 Characteristics of included studies

**Figure 1:**
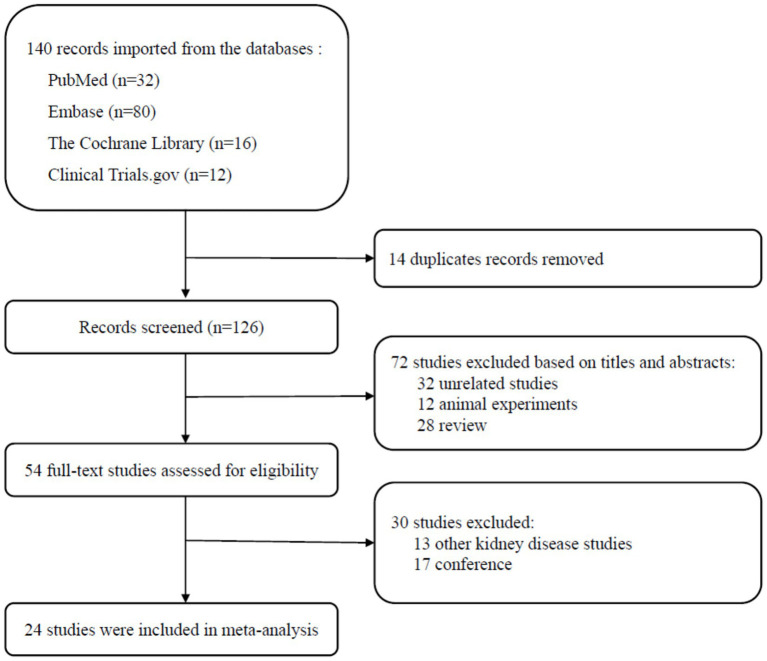
Search strategy diagram.

A total of 140 records were identified through the electronic search. After 126 titles and abstracts underwent screening, 54 articles underwent full-text review. This process culminated in the final inclusion of 24 articles (Figure 1).

The basic characteristics of the included studies are summarized in Table 1. All eligible studies were conducted by Chinese investigators and comprised one randomized controlled trial (RCT), representing the highest level of evidence, 14 retrospective observational studies, one prospective study, and eight illustrative case reports or case series. Across the included studies, treatment duration ranged from short-term follow-up of approximately 12–24 weeks to extended treatment periods exceeding 48 weeks in observational cohorts and case-based reports, allowing assessment of both early and sustained treatment responses. The enrolled patient populations spanned a broad clinical spectrum, including adult and pediatric patients, as well as clinically challenging subgroups such as individuals with post-transplant recurrent IgA nephropathy or refractory IgAN. Although weekly subcutaneous administration was the most commonly reported dosing schedule, telitacicept regimens varied across studies with respect to starting dose (e.g., 80, 160, or 240 mg), weight-based dosing strategies, and dose tapering or extension to every 2 weeks in selected patients.

**Table 1:** Summary of clinical studies evaluating telitacicept in patients with IgA nephropathy.

First author	Year	Country	Study type	Sample size (*n*)	Male/Female	Intervention in experimental group	Control group intervention	Duration	Outcome measures
Suojian Zhang (43)	2025	China	Case Report	1	0/1	A subcutaneous injection of telitacicept 160 mg weekly, and oral methylprednisolone 20 mg/day and valsartan 80 mg/day.	NA	24 weeks	Scr, Alb, 24hUPQ, UACR, and URBC.
Xueqing Ma (23)	2025	China	Retrospective Observational Study	54	31/23	Weekly subcutaneous injection of telitacicept (80 mg for weight 20-40 kg, 160 mg for >40 kg) combined with conventional therapy.	Conventional therapy	36 weeks (primary), up to 48 weeks follow-up	UPCR, 24hUPQ, Alb, eGFR, serum immunoglobulins (IgA, IgG, IgM), glucocorticoid dosage.
Lingqiu Dong (16)	2025	China	Prospective Observational Case–Control Study	63	13/50	Weekly subcutaneous injection of telitacicept 240 mg, combined with standard supportive therapy.	Supportive therapy: RASi/SGLT2i/HCQ; Immunosuppressive therapy: prednisone ± MMF	12 weeks (primary endpoint)	24hUPQ, eGFR.
Jiaojiao Liu (18)	2025	China	Retrospective Multicenter Study	16	7/9	Addition of weekly subcutaneous injection of telitacicept (dose unspecified) to existing background therapy.		Median 24 weeks follow-up (up to 48 weeks)	24hUPQ, UPCR, Alb, eGFR, immunoglobulins, CD19 + B cells.
Hui Li (32)	2025	China	Retrospective Single-Center Study	33	14/19	Initial weekly subcutaneous injection of telitacicept 160 mg, could be reduced to 80 mg/week upon remission, combined with conventional therapy.	Conventional therapy	24 weeks	24hUPQ, Scr, eGFR, complete remission rate (CR), partial remission rate (PR).
Yanyan Jin (24)	2025	China	Retrospective Study	12	9/3	Weekly subcutaneous injection of telitacicept (2.5 mg/kg, max 160 mg) combined with ACEi.		Median 36 weeks follow-up (up to 48 weeks)	24hUPQ, URBC, eGFR, Alb.
Qinjie Weng (14)	2025	China	Retrospective Study	82	48/34	Telitacicept 240 mg weekly (Short-term group: 6 months; Extended group: after 6 months, switched to once every 2 weeks until 9–12 months) combined with ACEi/ARB.	ACEi/ARB	48 weeks	24hUPQ, eGFR
Lin Tao (17)	2025	China	Retrospective Study	24	16/8	Group A: Telitacicept 160 mg/week monotherapy; Group B: Telitacicept + low-dose prednisone; Group C: Telitacicept + full-dose prednisone/immunosuppressant.		At least 12 weeks, median 22, up to 64 weeks	24hUPQ, eGFR, serum immunoglobulins, adverse events.
Lijun Liu (25)	2025	China	Retrospective Multicenter Study	97	44/53	Telitacicept (starting at 80 or 160 mg/week), 61.9% combined with glucocorticoids/immunosuppressants.		Median 12 weeks follow-up (up to 24 weeks)	24hUPQ, eGFR, serum immunoglobulins.
Jingyi Wu (19)	2025	China	Retrospective Study	15	10/5	Weekly subcutaneous injection of telitacicept 80 or 160 mg.		Median 25 weeks follow-up (up to 48 weeks)	24hUPQ, eGFR, Alb, hemoglobin.
Xue Shen (33)	2025	China	Cohort Study	677	313/364	Combination therapy including endothelin receptor antagonist, Nefecon, SGLT2i, hydroxychloroquine, or telitacicept.		Median 230 weeks follow-up	eGFR decline ≥50% or ESKD.
Shuang-xi Li (44)	2025	China	Case Report	2	1/1	Telitacicept 160 mg weekly ± glucocorticoids/mycophenolate mofetil (MMF).		24–36 weeks	24hUPQ, Scr, eGFR.
Shucheng Chen (30)	2025	China	Case Report	1	1/0	Telitacicept 160 mg weekly (later reduced to 80 mg weekly, then 80 mg every 2 weeks).	NA	20 weeks	24hUPQ, Alb, UACR, Scr.
Zishu Yuan (27)	2025	China	Case Report	1	1/0	Telitacicept 160 mg weekly, plus prednisone acetate 40 mg daily (later gradually tapered and discontinued).	NA	180 weeks	24hUPQ, Alb, eGFR, serum IgA/IgG/IgM.
Yanyan Jin (29)	2025	China	Retrospective Study	7	2/5	Subcutaneous injection of telitacicept once weekly (<40 kg: 80 mg; ≥40 kg: 160 mg).	NA (Self-controlled before and after)	Median 36 weeks follow-up	24hUPQ, URBC.
Jing Wang (28)	2025	China	Case Series	7	6/1	Weekly subcutaneous injection of telitacicept (80 or 160 mg) as add-on therapy to standard care (glucocorticoids ± other immunosuppressants).		24 weeks	24hUPQ, CD19 + B cells, IgA, IgM, Alb, complement C4, etc.
Lichen Xu (21)	2025	China	Retrospective Cohort Study	10	6/4	Patients with recurrent IgAN post-kidney transplant: Telitacicept added to baseline immunosuppressive maintenance (tacrolimus + mycophenolate + corticosteroids). Initial dose 160 mg/week, adjustable to 240 mg/week.		48 weeks	24hUPQ, Scr, eGFR, URBC.
Yangyang He (15)	2025	China	Retrospective, Multicenter Cohort Study	104	47/57	Telitacicept (160–240 mg/week) combined with low-dose corticosteroids.	MMF (1.0–1.5 g/day) combined with low-dose corticosteroids.	48 weeks	24hUPQ, eGFR, complete remission rate (CR), partial remission rate (PR), adverse events.
Huapan Shu (20)	2025	China	Retrospective Cohort Study	24	10/14	Telitacicept (160 mg/week, reduced after 6 months based on response) combined with low-dose MMF (1 g/day, reduced after 1 year).		Median 92 weeks follow-up	24hUPQ, eGFR, URBC, Scr.
Yan Shen (31)	2024	China	Case Report	1	0/1	Subcutaneous injection of telitacicept 160 mg/week, combined with MMF and glucocorticoids.	NA	24 weeks	24hUPQ, UACR, Alb, Scr, eGFR.
Meng Wang (26)	2024	China	Retrospective Case–Control Study	42	23/19	Telitacicept ± immunosuppressants.	Conventional immunosuppressive therapy	24 weeks	24hUPQ, eGFR.
Liping Sun (22)	2024	China	Case Report	3	2/1	Case 1: telitacicept 240 mg weekly for 12 weeks, then 160 mg weekly for 8 weeks, then 80 mg weekly; plus oral prednisone 20 mg/day; Case 2: telitacicept 240 mg weekly for 12 weeks, then 160 mg weekly, then 80 mg weekly; plus oral prednisone (tapered from 30 mg/day); Case 3: telitacicept 240 mg weekly for 12 weeks, then 160 mg weekly for 8 weeks, then 80 mg weekly; plus oral prednisone 21 mg/day.	NA	20 weeks	24hUPQ, Scr, Alb.
Xinru Du (45)	2024	China	Case Report	1	0/1	Telitacicept 80 mg weekly, plus oral prednisone acetate (initial dose unspecified, later gradually tapered and discontinued).	NA	32 weeks	24hUPQ, Scr, URBC.
Jicheng Lv (13)	2023	China	RCT	44	23/21	Telitacicept 160 mg or 240 mg weekly.	Placebo	24 weeks	24hUPQ, eGFR.

### 3.2 Efficacy outcomes

#### 3.2.1 Reduction of proteinuria: potent and clinically meaningful efficacy

As a key surrogate endpoint for assessing therapeutic response in IgAN, reduction in proteinuria was consistently and robustly demonstrated across all studies included in this review. One RCT conducted by Lv et al. (13) provided high-level evidence supporting the efficacy of telitacicept, demonstrating a significant 49% reduction in 24hUPQ excretion at 24 weeks compared with placebo, while eGFR remained stable. This landmark finding was subsequently corroborated by multiple real-world studies. For example, Weng et al. (14) reported that patients receiving extended telitacicept therapy for 12 months achieved a mean reduction in 24hUPQ of 56.8% from baseline. This magnitude of improvement stands in sharp contrast to the minimal change observed in a matched cohort receiving optimized supportive care with angiotensin-converting enzyme inhibitors or angiotensin II receptor blockers (ACEi/ARB) alone (0.3%). Importantly, a clear duration–response relationship was identified, with extended treatment demonstrating significantly greater efficacy than short-term intervention (28.6% reduction in 24hUPQ).

Compared with existing standard immunosuppressive regimens, telitacicept also exhibited clear therapeutic advantages. In a multicenter cohort study (15) involving 104 patients, the mean reduction in proteinuria after 12 months was significantly greater in the telitacicept group than in the MMF group (62.5% vs. 52.9%; *P* < 0.05). Moreover, the complete remission rate was significantly higher among patients treated with telitacicept (33.3% vs. 16.1%; *P* < 0.05) (15). Consistent with these findings, Dong et al. (16) reported an early and substantial treatment effect, with a 54.6% reduction in 24hUPQ observed as early as 12 weeks. This effect was comparable to that of conventional immunosuppressive therapy and markedly superior to supportive care alone.

Notably, telitacicept induced consistently high and sustained response rates across diverse patient populations. Tao et al. (17) reported an overall response rate (ORR) of 87.5%, while a pediatric cohort study demonstrated a complete remission rate of 62.5% in telitacicept-treated patients (18). Longitudinal data shows that the therapeutic benefit deepens with the prolongation of the treatment course, with a median decrease of 59.0% in proteinuria at 12 months of treatment (19), which can be further reduced to 87% at 24 months of treatment (20). Even in clinically challenging settings, such as recurrent IgAN following kidney transplantation, telitacicept demonstrated promising efficacy, with 60% of patients achieving a proteinuria reduction exceeding 30% within 6 months (21). Nevertheless, treatment response exhibited heterogeneity, particularly among patients with extremely high baseline proteinuria (>9 g/day), in whom efficacy varied substantially, suggesting that a heavy disease burden may attenuate therapeutic response (22).

It should be noted that most evidence supporting proteinuria reduction derives from retrospective observational studies and case-based reports, and the overall level of evidence remains limited.

#### 3.2.2 Renal function preservation: stability of eGFR and Scr

Preservation of renal function represents the ultimate therapeutic goal in IgAN management. This review found that telitacicept treatment was closely associated with stabilization of renal function. The majority of recent studies (16, 19, 20, 23–26) consistently reported stable eGFR levels throughout the treatment period. A quantitative comparative analysis by Weng et al. (14) further demonstrated that the annual rate of eGFR decline was slower in telitacicept treated patients than in those receiving standard care, suggesting a potential renoprotective effect. This stabilizing effect was also evident in high-risk populations. For instance, among patients with recurrent IgAN after kidney transplantation, Scr levels and eGFR remained stable in most individuals after 48 weeks of treatment (21). In a long-term case report, Yuan et al. (27) documented sustained renal function stability for up to 180 weeks.

However, it is worth noting that in the included studies, the term “renal function stability” was used descriptively to indicate the absence of a reported clinically meaningful decline in eGFR or increase in serum creatinine during the respective follow-up periods, rather than a uniform predefined threshold across studies.

#### 3.2.3 Improvements in other supportive biomarkers and clinical parameters

Beyond the primary endpoints, telitacicept therapy was associated with favorable changes in multiple secondary parameters related to disease pathophysiology, further supporting its proposed mechanism of action.

##### 3.2.3.1 Biomarker responses and mechanistic validation

An increase in serum albumin was reported in several studies (19, 23), but this finding more likely reflects reduced proteinuria and improved inflammatory status than direct nutritional improvement. From an immunological perspective, telitacicept effectively suppressed aberrant B-cell–mediated immune responses, evidenced by significant reductions in serum immunoglobulin levels (IgA, IgG, and IgM) as well as CD19^+^ B-cell counts (23, 28). In addition, amelioration of hematuria observed in pediatric and post-transplant populations (21, 29) provided further evidence of the drug’s capacity to attenuate inflammatory activity within the glomeruli.

##### 3.2.3.2 Glucocorticoid-sparing effect

A particularly notable clinical advantage of telitacicept is its pronounced glucocorticoid-sparing effect. Multiple studies (30, 31) reported that concomitant use of telitacicept enabled successful tapering or complete discontinuation of glucocorticoids. Quantitative analysis by He et al. (15) demonstrated that cumulative glucocorticoid exposure was significantly lower in the telitacicept group than in the MMF-treated group, potentially reducing the risk of long-term steroid related complications.

#### 3.2.4 Reporting of Cardiometabolic and nutritional endpoints

Although IgAN is increasingly recognized as a contributor to cardiometabolic burden through the cardiorenal axis, most included studies primarily focused on renal outcomes (proteinuria and eGFR). Cardiometabolic variables (e.g., blood pressure, lipid profile, uric acid, glycemic indicators, body weight/BMI, inflammatory markers) were variably defined and inconsistently reported, and formal cardiovascular endpoints were rarely prespecified. Likewise, documentation of structured lifestyle or nutritional interventions (e.g., dietary patterns, sodium restriction adherence, nutritional counseling, or metabolic optimization programs) was limited. This reporting gap precludes robust synthesis of the potential cardiometabolic and nutritional implications of telitacicept therapy and underscores the need for future trials to incorporate standardized cardiometabolic and nutrition-related outcomes.

### 3.3 Safety and tolerability profile

A pooled analysis of safety data from 24 studies indicated that telitacicept was generally well tolerated, with a manageable safety profile. The most frequently reported adverse events were mild to moderate injection-site reactions (including pain, erythema, and induration), which were common but typically transient and self-limiting (16, 32). The incidence of serious infections was low. A limited number of studies reported upper respiratory tract infections and reversible hypogammaglobulinemia (17, 18), most events were mild to moderate in severity and did not necessitate treatment discontinuation. Notably, a retrospective comparative study demonstrated a significantly lower overall incidence of adverse events in the telitacicept group than in the MMF group (22.9% vs. 42.9%; *p* = 0.032) (15). Across follow up periods extending up to 230 weeks, no treatment-related deaths or malignancies were reported, providing preliminary support for the long-term safety of telitacicept (33). Furthermore, its safety profile has been preliminarily validated in special populations, including pediatric patients and kidney transplant recipients (18, 21). No new or unexpected safety signals were consistently reported across study types.

Given the heterogeneity in study design and follow-up duration, adverse events reported in short-term randomized trials, retrospective cohorts, and long-term case reports are not directly comparable. Accordingly, safety findings in this review are summarized descriptively, and conclusions regarding long-term safety should be interpreted with caution.

## 4 Discussion

### 4.1 Telitacicept in IgAN: mechanism of action, efficacy, and safety

The available evidence indicates that telitacicept can substantially reduce proteinuria in patients with IgAN, with reported reductions ranging from 49 to 87%. In contrast to optimized supportive care comprising renin angiotensin aldosterone system (RAAS) blockade with angiotensin converting enzyme inhibitors or angiotensin II receptor blockers (ACEi/ARBs), blood pressure control, and lifestyle interventions -or conventional immunosuppressive strategies such as MMF, which primarily target downstream inflammatory pathways or exert nonspecific immunosuppression, telitacicept represents a targeted upstream immunomodulatory therapy.

Telitacicept is a recombinant human transmembrane activator and calcium modulator and cyclophilin ligand interactor (TACI)–Fc fusion protein that functions as a dual inhibitor of B-cell activation. By mimicking the extracellular domain of the TACI receptor, telitacicept binds with high affinity to and neutralizes excessive circulating BAFF/BLyS and APRIL (34, 35). Through this dual-target mechanism, telitacicept broadly inhibits B-cell development, maturation, and differentiation, while suppressing plasma cell survival. This results in reduced production of Gd-IgA1 and its associated pathogenic immune complexes (16, 36). Consequently, immune-complex deposition within the glomeruli is attenuated, representing a key upstream pathway through which telitacicept exerts therapeutic effects in IgAN. These mechanistic distinctions provide a biological rationale for the superior proteinuria-lowering efficacy of telitacicept compared with traditional treatment approaches (37).

Beyond proteinuria reduction, multiple studies have reported attenuation of eGFR decline in patients receiving telitacicept, suggesting a potential benefit on eGFR slope and a possible renoprotective effect. Given that approximately 40% of patients with IgAN progress to kidney failure within 20 years (13, 16, 19, 25), an intervention capable of delaying progression to end-stage kidney disease would have major implications for long-term outcomes. However, current data on the effect of telitacicept on hard kidney endpoints (including progression to kidney failure or sustained decline in eGFR) remain limited, and further long-term studies are required to clarify the durability and magnitude of this potential benefit.

Recent studies (15, 30) have demonstrated that telitacicept therapy facilitates a reduction in exposure to systemic glucocorticoids, thereby partially reshaping the traditional paradigm of long-term steroid dependence in IgAN management. By reducing cumulative glucocorticoid exposure, the risk of treatment-related adverse effects such as osteoporosis, diabetes mellitus, and infection may also be mitigated (37). These findings align with the evolving KDIGO framework, which emphasizes targeted immunomodulatory therapies as potential alternatives to nonspecific immunosuppression in glomerular diseases (38).

The efficacy of telitacicept has been consistently demonstrated across clinical trials and real-world observational studies. For example, Weng et al. (14) reported that after 12 months of treatment, the reduction in proteinuria was significantly greater in the telitacicept group than in patients receiving optimized supportive care alone with ACEi/ARBs (56.8% vs. 0.3%), highlighting the robustness and durability of its antiproteinuric effect. Importantly, telitacicept has also shown therapeutic promise in special populations with limited treatment options. In pediatric patients, complete remission rates of up to 62.5% have been reported (18), while response rates of approximately 60% have been observed in patients with post-transplant recurrent IgAN (21), suggesting that its efficacy may extend across diverse clinical contexts.

With respect to safety, telitacicept has generally demonstrated good tolerability in patients with IgAN. Available evidence indicates that the overall incidence of adverse events is comparable to that observed in control groups receiving standard of care, with no significant increase in serious adverse events. Nevertheless, certain potential risks warrant careful consideration. Although hypogammaglobulinemia has been reported in some studies (18, 25), it was typically mild and did not necessitate treatment discontinuation; nonetheless, long-term monitoring of immunoglobulin levels is advisable. Moreover, data on long-term safety beyond 5 years remain insufficient, underscoring the need for extended follow-up.

Despite encouraging evidence, several controversies and challenges remain. Sun et al. (22) observed a diminished therapeutic response in patients with very high baseline proteinuria (>9 g/day), suggesting that patients at higher risk (such as those with severe disease burden or persistent proteinuria despite optimized supportive care) may derive less benefit from telitacicept. In addition, evidence remains limited regarding optimal dosing strategies, long-term efficacy, cost-effectiveness, and differential responses across patient subgroups, highlighting the need for further investigation to refine clinical implementation. By achieving sustained proteinuria reduction and stabilizing renal function, telitacicept may indirectly support cardiometabolic homeostasis, thereby creating favorable conditions for downstream nutritional and lifestyle-based interventions emphasized in integrated cardiometabolic disease management.

### 4.2 Nutritional strategies and Cardiometabolic management in IgAN

IgAN is increasingly viewed as a systemic condition in which chronic inflammation, persistent proteinuria, and progressive renal dysfunction contribute to cardiometabolic risk via the cardiorenal axis (39). Accordingly, nutritional and lifestyle-based strategies remain foundational components of long-term risk reduction, particularly for blood pressure control, metabolic optimization, and cardiovascular prevention. In clinical practice, dietary sodium restriction, adoption of cardioprotective dietary patterns (e.g., DASH-style or Mediterranean-style approaches), weight management, and individualized counseling on protein intake and phosphorus burden (especially in patients with reduced kidney function) are commonly recommended as part of comprehensive care (38, 40).

However, the effectiveness and sustainability of these strategies may be constrained in patients with active immune-driven renal injury and persistent proteinuria. Ongoing proteinuria is associated with edema, reduced exercise tolerance, and higher risk of cardiovascular complications, which can undermine long-term adherence to dietary and lifestyle programs (41). Moreover, reliance on systemic glucocorticoids may worsen cardiometabolic profiles (e.g., glycemic control, weight gain, dyslipidemia), thereby counteracting lifestyle-based prevention efforts (42).

In this context, upstream immunomodulatory therapies that stabilize renal disease activity may indirectly support cardiometabolic risk management. The evidence summarized in this scoping review suggests that telitacicept consistently reduces proteinuria and is associated with stable kidney function across heterogeneous study designs. Improvements in serum albumin reported in several studies are more plausibly interpreted as reflecting reduced urinary protein loss and attenuated inflammatory burden rather than direct nutritional effects (41). Additionally, the observed glucocorticoid-sparing potential may have clinically meaningful implications for cardiometabolic risk mitigation by reducing steroid-associated metabolic adverse effects (42).

Importantly, direct evidence linking telitacicept to cardiometabolic outcomes (e.g., blood pressure, lipid profile, glycemic indicators, body weight/BMI, inflammatory markers) or to the effectiveness of structured nutritional interventions remains limited due to inconsistent reporting and the lack of prespecified endpoints. Future randomized and pragmatic studies should incorporate standardized cardiometabolic and nutrition-related outcomes, document background dietary counseling and adherence, and evaluate potential synergy between pharmacologic immunomodulation and structured nutritional/lifestyle programs. Such designs would clarify whether stabilizing proteinuria and renal function can meaningfully facilitate long-term nutritional strategies and improve cardiorenal outcomes in high-risk IgAN populations.

### 4.3 Limitations of current evidence

Although the available data are clinically informative, several limitations must be acknowledged. First, only one RCT (13) has been reported to date, while the majority of the available evidence is derived from retrospective observational studies, case reports, and small case series, which are inherently susceptible to selection and information biases. In keeping with the aims of a scoping review, case-based evidence was intentionally included to map early clinical experience, capture rare or special clinical scenarios, and identify potential signals of efficacy or safety. However, such evidence should be interpreted with caution and regarded as descriptive and hypothesis-generating rather than confirmatory, and should not be considered equivalent to data derived from randomized controlled trials or well-designed observational studies. Second, sample sizes in most studies have been relatively small (median approximately 60 patients), limiting statistical power and generalizability. Larger, well-designed prospective studies are therefore required to validate current findings and to better characterize the efficacy and safety profiles of telitacicept across different patient populations. In addition, data on long-term outcomes beyond 5 years and formal pharmacoeconomic evaluations remain scarce.

### 4.4 Future research directions

Based on current evidence and aligned with KDIGO priorities, future research should focus on the following areas: (1) Large-scale RCTs with hard clinical endpoints (such as kidney failure, sustained decline in eGFR, and all-cause mortality) to definitively establish the efficacy and safety of telitacicept, including head-to-head comparisons with optimized supportive care and existing standard therapies; (2) Identification of biomarkers predictive of treatment response, including serum BAFF levels, Gd-IgA1 titers, and genetic polymorphisms (e.g., HLA-DQB1), integrated with validated risk prediction tools to enable individualized therapy; (3) Further mechanistic studies elucidating the effects of telitacicept on Gd-IgA1 glycosylation, mesangial cell signaling pathways, and renal fibrogenesis; (4) Establishment of long-term, registry-based real-world studies to assess sustained effectiveness, safety, and cost-effectiveness across diverse clinical settings; (5) Exploration of combination strategies incorporating telitacicept with other therapies (such as sodium glucose cotransporter 2 inhibitors, ACEi/ARB, or MMF) to identify potential synergistic effects and define optimal treatment algorithms.

## 5 Conclusion

Current evidence suggests that telitacicept, a dual BAFF/APRIL inhibitor, may represent a promising targeted therapeutic option for IgA nephropathy. Available studies consistently report clinically meaningful proteinuria reduction, generally stable kidney function during follow-up, and an acceptable safety profile. Given the cardiorenal link between persistent proteinuria and cardiometabolic risk, these effects may support broader cardiometabolic prevention and management strategies, including nutrition- and lifestyle-based interventions, in clinical practice. However, the evidence is largely observational and based on surrogate endpoints, and long-term outcomes and optimal patient selection remain uncertain. Future well-designed randomized trials with biomarker-guided approaches and long-term renal and cardiometabolic endpoints are needed.
