# Supplementary material for: Immune-mediated renal injury and cardiometabolic risk in IgA nephropathy: clinical evidence on telitacicept from a scoping review
Source: Front Nutr. 2026 Mar 17;13:1790988. doi: 10.3389/fnut.2026.1790988 (PMC13036128; doi:10.3389/fnut.2026.1790988)
Supplement: Supplementary file 1 [file supplementary_file_1.docx]

**PubMed**

#1 "Telitacicept"[Title/Abstract] OR "RC18"[Title/Abstract] OR "TACI-Fc"[Title/Abstract]

#2 "IgA Nephropathy"[Mesh] OR "Glomerulonephritis, IGA"[Mesh]

#3 "Berger‘s Disease"[Title/Abstract] OR "Immunoglobulin A Nephropathy"[Title/Abstract] OR "IgAN"[Title/Abstract]

#4 #2 OR #3

#5 #1 AND #4

**Embase**

#1 ‘telitacicept‘/exp OR ‘telitacicept‘:ti,ab,kw OR ‘rc18‘:ti,ab,kw OR ‘taci-fc‘:ti,ab,kw

#2 ‘iga nephropathy‘/exp

#3 ‘berger disease‘:ti,ab,kw OR ‘immunoglobulin a nephropathy‘:ti,ab,kw OR ‘igan‘:ti,ab,kw

#4 #2 OR #3

#5 #1 AND #4

**the Cochrane Central Register of Controlled Trials**

#1 (iga nephropathy):ti,ab,kw OR (Immunoglobulin a Nephropathy):ti,ab,kw OR (immunoglobulin a nephropathy):ti,ab,kw OR (iga glomeruloneph):ti,ab,kw OR (berger disease):ti,ab,kw OR (IgAGN):ti,ab,kw OR (igAN):ti,ab,kw

#2 (Telitacicept):ti,ab,kw OR (RC18):ti,ab,kw OR (TACI-Fc):ti,ab,kw

#9 #1 AND #2

**ClinicalTrials.gov**

Intervention/treatment: Telitacicept OR RC18 OR TACI-Fc

Condition/disease: iga nephropathy OR Immunoglobulin a Nephropathy OR immunoglobulin a nephropathy OR iga glomeruloneph OR berger disease OR IgAGN OR IgaN
